# Escaping from CRISPR–Cas-mediated knockout: the facts, mechanisms, and applications

**DOI:** 10.1186/s11658-024-00565-x

**Published:** 2024-04-08

**Authors:** Ying Wang, Yujing Zhai, Mingzhe Zhang, Chunlin Song, Yuqing Zhang, Gang Zhang

**Affiliations:** 1 The Cancer Institute, The Affiliated Hospital of Qingdao University, Qingdao University, Qingdao, China; 2 School of Public Health, Qingdao University, Qingdao, China

**Keywords:** CRISPR, Knockout escaping, Alternative splicing, Translation reinitiation

## Abstract

Clustered regularly interspaced short palindromic repeats and associated Cas protein (CRISPR–Cas), a powerful genome editing tool, has revolutionized gene function investigation and exhibits huge potential for clinical applications. CRISPR–Cas-mediated gene knockout has already become a routine method in research laboratories. However, in the last few years, accumulating evidences have demonstrated that genes knocked out by CRISPR–Cas may not be truly silenced. Functional residual proteins could be generated in such knockout organisms to compensate the putative loss of function, termed herein knockout escaping. In line with this, several CRISPR–Cas-mediated knockout screenings have discovered much less abnormal phenotypes than expected. How does knockout escaping happen and how often does it happen have not been systematically reviewed yet. Without knowing this, knockout results could easily be misinterpreted. In this review, we summarize these evidences and propose two main mechanisms allowing knockout escaping. To avoid the confusion caused by knockout escaping, several strategies are discussed as well as their advantages and disadvantages. On the other hand, knockout escaping also provides convenient tools for studying essential genes and treating monogenic disorders such as Duchenne muscular dystrophy, which are discussed in the end.

## Introduction

CRISPR–Cas refers to the adaptive immune system in prokaryotes. It stores part of the invader’s DNA information into the genomic CRISPR array. This small piece of DNA can be transcribed into a guide CRISPR RNA (crRNA), which forms a complex with Cas protein with nuclease activity. During a second infection, the foreign DNA can be recognized by this guide crRNA and degraded by the associated Cas nuclease [1]. Inspired by this elegant system, several groups successfully applied it for genome editing in human cells in 2013, which initiated a new era for both basic research and clinical applications [2–4]. Now, CRISPR–Cas is more often referred to as a powerful genome editing method composed of a programmable single-guide RNA (sgRNA) that specifically targets the associated Cas protein onto genomic loci via RNA–DNA complementarity and the Cas protein that performs editing on site via its nuclease activity or engineered novel activity. On the basis of the original CRISPR–Cas system, numerous new genome editing tools have been developed for different purposes such as gene knockout, transcriptional regulation, multiplexed editing, site-specific modifications, genetic sequence insertions, etc. [5]. In this review, only CRISPR–Cas-mediated gene knockout is discussed.

Gene knockout is probably the most common application of the CRISPR–Cas system. Guided by the sgRNA, the Cas protein locates on the intended genomic site and cleaves the double DNA strands. The double-stranded break could be repaired by error-prone nonhomologous end-joining (NHEJ), which frequently leads to random DNA insertions or deletions (indels). When the indels are within coding exons and not multiplicity of three, the open reading frame (ORF) is shifted and premature termination codon (PTC) occurs as a result. PTC further induces nonsense-mediated decay of the mutant mRNA (NMD), an mRNA quality control mechanism to prevent potentially toxic polypeptides [6].

Numerous knockout cell lines or organisms have been generated by CRISPS–Cas during the last decade, which has significantly promoted gene function investigations. Surprisingly, conflicting results have repeatedly been observed between forward and reverse genetic approaches; For example, poor correlation of the essential genes identified with shRNA silencing and CRISPR–Cas has been reported in human cells [7]. A similar observation was also made in zebrafish [8, 9]. This discrepancy was proposed to result from the off-target effect by RNA inteference or genetic compensation within the organisms. However, the last few years have witnessed many cases showing the presence of residual proteins from knocked-out genes that were able to partially or even fully rescue the loss of function. We name this knockout escaping, and it is reasonable to believe that this discrepancy could also come from knockout escaping. Here, we summarize these evidences and highlight a few studies including concrete function analysis of the residual protein in knockout cells or organisms. We also discuss the mechanisms behind knockout escaping and propose that translation reinitiation and alternative splicing, but not nonsense-associated alternative splicing (NAS), are its main sources. To get rid of the residual protein and avoid knockout escaping, several strategies are suggested in the following section. Though knockout escaping could cause severe problems regarding phenotype interpretation, it may also facilitate gene function investigations and clinical applications, as demonstrated by the examples in the final part of this review.

## Evidence for knockout escaping

To the best of the authors’ knowledge, the first report showing the existence of in-frame transcript and possible residual protein of the targeted gene was a study on *CDC14*. Uddin and colleagues were studying human *CDC14* gene, whose homolog in budding yeast plays essential roles in cell division. They knocked out *CDC14A* and *CDC14B* in cell lines by inserting a large DNA fragment containing a PTC and a selection marker into the targeted exons with the help of either zinc-finger nuclease (ZFN) or CRISPR–Cas9. Surprisingly, mRNA analysis did not find the inserted DNA fragment in either case owing to unexpected exon skipping. Since the nucleotide number of the skipped exon was dividable by three, the reading frame remained intact and the internal deleted protein of CDC14A and CDC14B was likely produced in the knockout cells. The truncated CDC14B protein was predicted to be functional as the small deletion was outside of the phosphatase domain. Owing to the lack of proper antibodies, this was not confirmed [10]. Since then, more than 20 studies have identified in-frame transcripts or truncated proteins from knockout organisms generated by the CRISPR–Cas system (Table 1) [10–37]. One interesting study from the Farber group provided direct evidence explaining the low frequency of mutant phenotypes by reverse genetics in zebrafish. Among seven fish lines containing either point mutations introduced by chemical mutagenesis or indels introduced by CRISPR–Cas9, alternative splicing occurred in six lines, resulting in in-frame transcripts in three of them. The protein products from these in-frame transcripts may still preserve the gene function and account for the lack of mutant phenotype [16].

**Table 1:** Reported cases with alternative splicing, exon skipping, and translation reinitiation in knockout models generated by CRISPR–Cas

Working model	Targeted gene	In-frame transcript	Truncated protein	Function analysis	Mechanisms proposed	Ref.
HCT116; RPE1	*CDC14A*	Detected	Not examined	Not examined	Exon skipping	[10]
HeLa; HEK293T	*FLOT1*; *AGA*	Detected	Detected	Not examined	Exon skipping	[11]
NIH3T3	*Gli3*	Not examined	Detected	Not examined	Translation reinitiation	[12]
zebrafish	*chd7*; *hace1*; *pycr1a*	Not detected	Predicted for *hace1*, *pycr1a*	Functional for hace1	Exon skipping; translation reinitiation	[13]
teloHAEC	*PHACTR1*	Detected	Detected	Not examined	Exon skipping	[14]
KP; NIH3T3; C2C12; HCT116	*Kras*; *Ctnnb1*; *Dmd*; *LMNA*; *p65*	Detected for *Ctnnb1*, *Dmd*, *LMNA*	Detected for *Ctnnb1*	Functional for *Ctnnb1*	Exon skipping	[15]
Zebrafish	*smyd1a*	Not detected	Not examined	Not examined	Alternative splicing	[16]
Rabbit	*DMD*; *LMNA*; *GCK*; *ANO5*; *GHR*	Not detected	Not examined	Not examined	Alternative splicing	[17]
Locust	*LmigOr35*	Detected	Not examined	Not examined	Exon skipping	[18]
RPE1	*BUB1*	Detected	Detected	Functional	Alternative splicing	[19]
Mouse	*Ccnb3*	Detected	Detected	May be functional	Alternative splicing	[20]
HeLa	*BUB1*	Not examined	Detected	Functional	Not proposed	[21]
HAP1; K562	*136 genes*	Examined and detected in *BRD3*, *MTF2*, *PI4KB*, *DNMT1*, *NGLY1*	Detected in ~ 30% knock out cell lines	Examined *BRD4*, *DNMT1*, *NGLY1* and functional	Exon skipping; translation reinitiation	[22]
HAP1; RMS13; MIA	*RICTOR*; *VPS35*; *TOP1*; *SIRT1*; *CTNNB1*; *AXIN1*; *LRP6*; *PTEN*; *TBK1*; *BAP1*; *TLE3*; *PPM1A*; *BCL2L2*; *LPR5*; *SUFU*; *LKB1*	Detected in *TOP1*, *LKB1*, *SUFU*	Detected in *TOP1*, *SIRT1*, *CTNNB1*, *LRP6*, *LKB1*, *LRP5*	Examined *TOP1*, *LRP5* and functional	Exon skipping; translation reinitiation	[23]
Rice	*OsIAA23*	Detected	Not examined	Functional	Alternative splicing	[24]
C2C12	*CK2α*; *CK2α′*; *CK2β*	Not annotated	Detected	Functional	Not proposed	[25, 26]
Mouse	*Rhbdf1*	Detected	Not examined	Functional	Translation reinitiation	[27]
BEAS-2B; A549; MDA-MB-231	*MDIG*	Detected	Detected	Not examined	Exon skipping	[28]
Mouse	*Ets2*	Detected	Detected	Functional	Exon skipping	[29]
HeLa	*CDC20*	Detected	Detected	Functional	Translation reinitiation	[30]
HT29	*EpCAM*	Detected	Detected	Functional	Exon skipping; translation reinitiation	[31]
Rice	*WDA1*; *BC10* and another 71 genes	Detected in 39 knockouts	Examined in *WDA1* and detected	Examined in *WDA1*, *BC10* and functional	Exon skipping; alternative splicing	[32]
THP1	*IFNAR2*	Detected	Detected	Functional	Exon skipping	[33]
HL1	*PKP2*; *DSG2*; *DSC2*; *DSP*; *JUP*	Detected in *DSP*, *JUP*	Detected in *DSP*, *JUP*	May be functional for *DSP*	Translation reinitiation	[34]
A375	*XPA*	Detected	Detected	Functional	Alternative splicing; translation reinitiation	[35]
A549; H1703	*NRF2*	Detected	Detected	Functional	Exon skipping	[36]
Mice	*Ctnnb1*	Detected	Detected	Functional	Exon skipping	[37]

Studies on the kinase CK2 demonstrated how the residual protein could mislead researchers [25, 26, 38]. Composed by two catalytic subunits α and α′ together with a dimer of β subunit, CK2 is involved in multiple signal pathways and associates with a panel of diseases, especially cancer. It is known that a lack of CK2 causes embryonic lethality. To understand whether CK2 is also essential in cells, Borgo and colleagues generated knockout cell lines by targeting the subunit genes using CRISPR–Cas9. The cell lines with both α and α′ disrupted displayed minimal kinase activity toward CK2 substrates, indicating that CK2 is dispensable for cell viability [38]. However, this conclusion was questioned later as the authors noticed that the phosphorylation of another CK2 substrate CDC37 S13 was only partially reduced in the double-knockout cell lines. pS13 signals could be further reduced by CK2 inhibitors, indicating that residual CK2 activity remained in these cells. By using a new antibody against CK2α′, the authors detected a faint band running slightly faster than the wild-type protein by western blot, which was not detected in their previous study. Further analysis showed that an N-terminal truncated CK2α′ was produced in the double-knockout cells, which can bind the β subunit and maintain a low kinase activity. The low activity may be sufficient for cell survival, but not for cell differentiation and transformation [25, 26]. Obviously, the essentiality of CK2 in cells remains unclear at the moment.

**Fig. 1:**
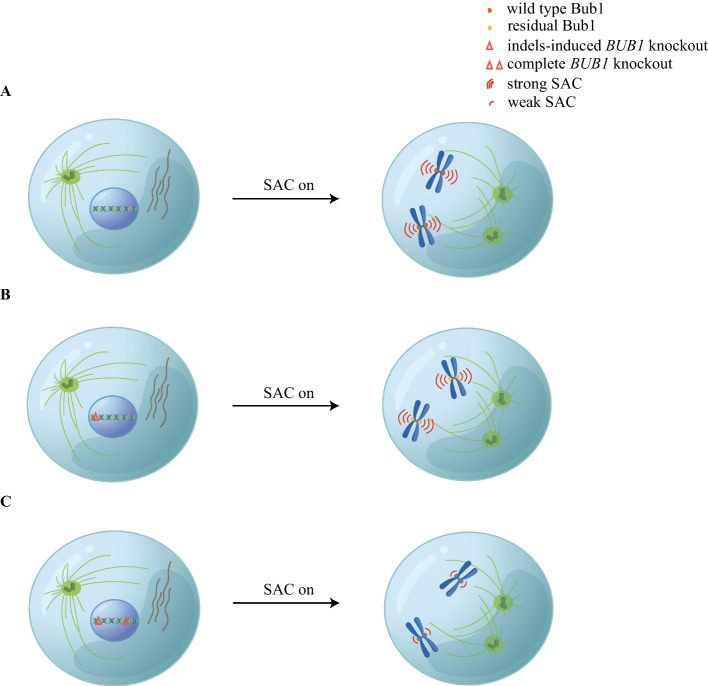
Misinterpretation of *BUB1* function owing to knockout escaping. **A** The wild-type cell shows strong SAC when activated. **B** The *Bub1* knockout cell generated via frame shifting induced by CRISPR–Cas produces residual Bub1 protein, which fully supports SAC activity. **C** The complete *Bub1* knockout cell, like through physical removal of the DNA fragment of *BUB1* by generating two double-strand breaks flanking the gene, could only maintain low SAC activity

One more similar case came from studies on the mitotic checkpoint protein Bub1. *BUB1* was one of the first mitotic checkpoint genes identified in the initial yeast screening [39, 40]. It is well established that Bub1 recruits checkpoint proteins such as BubR1, Mad1, Cdc20, and RZZ complex onto kinetochores and catalyzes the formation of the MCC complex, the inhibitor of E3 ligase APC/C [41]. Thus, a lot of confusion was induced when it was reported that the mitotic checkpoint was intact in *Bub1* knockout cells [19, 21, 42, 43]. To explain this controversial result, Rodriguez-Rodriguez et al. examined Bub1 signals by quantitative immunofluorescence and found around 3–30% of Bub1 on kinetochores in knockout cells compared with parental cells. Meanwhile, shorter transcripts with partial or whole exon 4 skipped were also detected in the knockout cells [19]. Another study conducted immunoprecipitation by antibodies against Bub1-interacting protein BubR1 or Bub3 in *Bub1* knockout cells and detected multiple Bub1 peptides by mass spectrometry. As estimated on the basis of peptide intensities, 2–8% of Bub1 was produced in these knockout cells, being sufficient for mitotic checkpoint activation [21] (Fig. 1).

A similar observation was also reported during the development of novel anticancer molecules. The gene *EpCAM* was disrupted by CRISPR–Cas9 in HT29 cells, but this did not abolish the sensitivity of the cells toward the EpCAM specific inhibitor. RT-PCR analysis showed the existence of an in-frame transcript without exon 2, which was translated into a functional protein with 36 amino acids missing to maintain the sensitivity [31].

Determining the frequency of occurrence of in-frame transcripts and residual proteins in knockout models is critical for evaluating the severity caused by knockout escaping. A collaboration among several groups assessed this issue using knockout HAP cells. They systematically examined 193 cell lines with 136 genes containing verified deletions by quantitative transcriptomics and proteomics. The mRNA levels of the mutated genes displayed wide variations, implying distinct NMD response to these transcripts. More strikingly, residual protein at levels from low to original was detected in one-third of the knockout cells. This number is obviously an underestimate, since in some cases the protein level could be lower than the detection limit. For example, around 60% of deglycosylation activity was maintained in *NGLY1* knockout cells without residual protein detected. In case such as this, one more step of protein enrichment by immunoprecipitation may help in detecting the protein. Functional analysis of three residual proteins including BRD4, DNMT1, and NGLY1 revealed partial functionality maintained in knockout cells [22]. A similar study on 13 HAP cell lines harboring frame-shifting indels identified altered mRNA splicing in six cell lines and residual proteins in four cell lines. One of the truncated proteins, TOP1, was still able to relax the supercoiled DNA [23].

Functional analysis has also been conducted in mice with genes knocked out, giving similar results as in cells or zebrafish [27, 29, 37]; For example, different gene knockout strategies were compared for the mutant phenotypes in mice, namely a definitive-null strategy using a bacterial artificial chromosome (BAC) to remove the entire genomic sequence of the target gene, a KO-first strategy that excised exons 4–11 flanked by the *loxP* sites with the help of Cre recombinase, and CRISPR–Cas9-mediated knockout targeting exon 2 and 3 of the *RHBDF1* gene. The phenotypes displayed by the knockout mice from the three strategies were strikingly different. The knockout mice from the definitive-null strategy died either by P14 or by 4 weeks, while the knockouts from the other two strategies were healthy and fertile. Further analysis found that CRISPR–Cas9-mediated knockout mice reinitiated translation from the next in-frame AUG, resulting in functional N-terminally truncated RHBDF1 protein to maintain the normancy of the knockout mice [27]. Another mouse study of the gene *Ets2* revealed that frame-shifting deletion induced exon 8 skipping and a functional truncated protein specifically expressed in skin [29]. Following their previous finding that targeting exon 3 of *Ctnnb1* resulted in exon skipping and in-frame transcript [15], Mou and colleagues studied the possibility of exon skipping in mice via tail vein injection of plasmid expressing Cas9 and sgRNA. RT-PCR detected a PCR band corresponding to the transcript with exon skipped, and immunohistochemistry also found a small number of hepatocytes with nuclear β-catenin, indicating the occurrence of exon skipping and residual protein expression in vivo [37].

All the above data were collected from mammalian cells or model animals, but does knockout escaping also occur in other species such as plants? Indeed, a recent study on rice examined the transcripts from mutant collections of 73 genes with frame-shifting indels induced by CRISPR–Cas9 and found that more than one-half of mutants had frame-restored transcripts. The authors further examined two mutants of *WDA1* and *BC10*, and found that these transcripts were able to produce functional proteins. Since these transcripts were generally in low abundance and the truncation may impair protein function to different extents, the authors assumed that a full rescue might be rare in rice [32].

On the basis of the above results, we speculate that residual protein expression could be a general byproduct of CRISPR–Cas-mediated gene knockout. A rough estimation from these studies indicates that at least 30–50% of knockout cells or organisms are producing altered transcripts and proteins. Though in some cases the residual protein was detected, in other cases it was completely invisible owing to low expression levels, the lack of available antibodies, the disruption of antigens, and limited detection methods. Ignorance of the residual proteins could lead to serious misinterpretation of knockout results, as described above.

## Mechanisms for knockout escaping

Though a complete understanding of the mechanisms for knockout escaping awaits more systematic investigations, several mechanisms have been suggested from the above studies (Table 1). We believe the knockout escaping could come from translation reinitiation and alternative splicing, as explained below.

## Translation reinitiation

It is estimated that around 50% of nonsense variants are degraded by NMD [44–46]. For those not degraded by NMD, N-terminus truncated proteins could be produced via translation reinitiation. Translation reinitiation occurs when a ribosome is not undergoing recycling and released from mRNA as the translation terminates, but starts translating a downstream ORF on the same mRNA. Translation reinitiation occurs frequently with PTC near the normal start codon and a downstream AUG codon in close vicinity. The distance between PTC and the nearby AUG may be a key determinant for the reinitiation efficiency. If the translation reinitiation starts from an in-frame AUG, an N-terminal truncated protein will be produced, which may maintain certain functionality [47, 48].

**Fig. 2:**
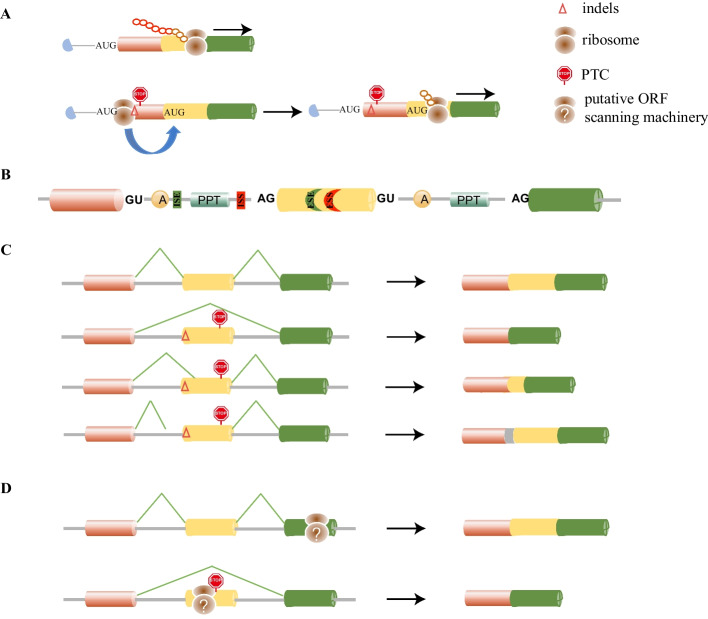
Mechanisms for the production of residual proteins in knockout cells. **A** CRISPR–Cas introduces indels and PTC in early exon. N-terminal truncated protein could be produced by translation reinitiation using another AUG downstream PTC. **B** *cis*-elements on pre-mRNA regulates splicing. Colored cylinders represent exons, and gray lines represent introns. *GU* 5′ splice site, *A* the branch site, *ISE* intronic splicing enhancer, *PPT* the polypyrimidine tract, *ISS* intronic splicing silencer, *AG* 3′ splice site, *ESE* exonic splicing enhancer, *ESS* exonic splicing silencer. **C** The indels induce alternative splicing by disrupting the original splicing motif and/or activating cryptic splicing motif. Green lines indicate the joining sites during the alternative splicing. New transcript may restore the reading frame and produce internal deleted/inserted protein. For simplicity reasons, only the case of indels and PTC in the same exon is shown here. **D** The putative ORF-dependent NAS model proposes an unidentified macromolecular machinery within the nucleus examining the pre-mRNA and inducing alternative splicing in the presence of PTC

A classic example is from the gene *ATRX* encoding a chromatin remodeling protein. Nonsense mutations in *ATRX* usually cause severe mental retardation. Intriguingly, patients with the nonsense mutation R37X only displayed mild mental disorder. Examination of the cells derived from the patients revealed the presence of functional truncated protein generated by translation reinitiation from the AUG downstream R37X, which partially rescued the severe null phenotype in the patients [49]. Regarding CRISPR–Cas-mediated knockouts, translation reinitiation downstream of PTCs may be a major reason for the production of the residual proteins [12, 13, 22, 23, 27, 30, 31, 34, 35] (Fig. 2A).

## Alternative splicing

The PTC-harboring transcripts could also be converted into ones without PTC through alternative splicing that excises the region containing PTC. Splicing is the process that converts a precursor mRNA (pre-mRNA) into mRNA, which relies on the spliceosome, a macromolecular protein–RNA complex, to recognize the intron/exon boundaries, remove introns, and join exons [50–54]. The precise assembly of the spliceosome on pre-mRNA requires interactions between the splicing *cis*-elements and their cognate *trans*-acting factors. The *cis*-elements refer to consensus sequences on pre-mRNA, including the essential ones such as the 5′ splice site, the branch point sequence (BPS), the polypyrimidine tract (PPT), and the 3′ splice site and auxiliary ones such as intronic splicing enhancers (ISEs) and silencers (ISSs), and exonic splicing enhancers (ESEs) and silencers (ESSs) (Fig. 2B). The *trans*-acting factors refer to ribonucleoproteins (RNPs) and RNA binding proteins (RBPs) associated with the spliceosome, which could recognize and bind to the *cis*-element through either the small nuclear RNAs within RNPs or the RNA binding motifs on RBPs. Any disturbance of the *cis*-elements or *trans*-acting factors may cause alternative splicing, leading to an altered exon combination via the usage of different splice sites (Fig. 2C). Here, we only discuss the effect on splicing by *cis*-element changes within exons, since CRISPR–Cas-mediated gene knockout normally targets exons.

Indels can directly disrupt the intron–exon boundaries or the exonic *cis*-elements, resulting in the failure of intron–exon boundary recognition (Table 1). In this case, the whole exon could be skipped, as illustrated in Fig. 2C. Note that indels may not completely stop intron–exon boundary recognition, thus multiple transcripts could be produced, including the full length-transcript harboring indels, which in many cases is still the major isoform. In other cases, indels generate new *cis*-elements or activate cryptic ones [16, 23, 28, 32]. As a result, a fragment of exon or intron may be excised or included in the final mRNAs, as shown in Fig. 2C. In addition, indels may also affect RNA secondary structure, which could induce alternative splicing [10, 23, 55]. In either case, if one of the final transcripts eliminates the PTC and restores the reading frame, it can be translated into a protein that may preserve the original functionality, albeit with a loss or gain of certain amino acids internally (Fig. 2C).

Nonsense-associated alternative splicing (NAS) refers to alternative splicing induced by PTC that can generate transcripts by skipping the disturbing PTC [56]. Two models have been proposed, including the motif-dependent model and ORF-dependent model [57–61]. Motif-dependent NAS refers to the PTC caused by nonsense mutation located within and disrupting the exonic *cis*-element [57–60]. As a result, alternative splicing is induced to produce a transcript with the exon containing the PTC excised. In this scenario, other mutations such as silent mutation or missense mutation will also cause altered splicing, although in some cases nonsense mutations are preferred. Regarding CRISPR–Cas-mediated gene knockout, PTC is generated through indel-induced frame shift, but not through nucleotide mutagenesis. Therefore, the PTC itself does not affect the *cis*-element, and the motif-dependent model does not apply here.

ORF-dependent NAS is highly controversial. It was proposed that, in the nucleus, a translation-like machinery existed and examined the ORF integrity of the pre-mRNA [61]. In case of an interrupted reading frame, alternative splicing was somehow induced, resulting in exon skipping and PTC removal (Fig. 2D). However, this theory was seriously challenged by the fact that the most compelling evidence could not be reproduced, either with the same gene TCR-β or with a similar gene Ig-µ [62–64]. The failure to identify the translation-like machinery in the nucleus after 20 years since the original proposal makes the ORF-dependent model even more questionable. Till now, more studies support the notion that nonsense mutations either disrupt the original splicing sites or generate new splicing sites, which results in alternative splicing [32, 59, 60, 65–78]. Regarding knockout escaping, one study in rabbit is in favor of ORF-dependent NAS for the observed exon skipping [17]. However, a thorough investigation to rule out other possibilities was missing, and no solid conclusion could be made from this study regarding the existence and involvement of ORF-dependent NAS in knockout escaping.

In summary, alternative splicing and translation reinitiation are very likely the main reasons accounting for most knockout escaping.

## Strategies to avoid knockout escaping

To avoid the interference of the residual proteins, a few methods could be considered when designing knockout strategies. Introducing two DNA breakages simultaneously will physically remove the DNA sequence in between the breakages on chromosomes [79, 80]. In this way, the cell will permanently lose the targeted gene. However, as shown by Hosur et al., permanent removal of several exons may not be enough to stop the production of functional truncated protein via translation reinitiation or alternative splicing from the remaining exons [27]. Therefore, physically removing the majority of the exons seems more promising. For this purpose, the type I CRISPR–Cas system, which enables long-range genome deletions, could be useful [81]. On the other hand, the loss of a large piece of DNA may affect other genes or gene transcription elements located within the removed DNA segment. For essential genes, no cell line or organisms could survive such a strategy.

Inducible gene excision such as the Cre-loxP recombination system could be another option. Though the method was originally designed for mice, efforts have been conducted to extend its application to human cells [82–84]. This system requires the knockin of two 34-bp loxP DNA motifs at desired positions flanking the target gene in cells expressing an inducible Cre recombinase. The generation of such a cell line could be time-consuming and high risk owing to the low knockin efficiency, but it could become a routine method in the future with improved knockin efficiency. The main advantage of this method is the ability to cultivate inducible knockout cell lines or organisms, which makes the study of essential genes much easier. However, this faces the same concerns as discussed above.

Alternatively, to generate a cell line stably expressing a single guide RNA targeting the most critical exon with inducible Cas9 protein is relatively easier than the above method [85]. By using this strategy, residual inactive protein could still be produced, but not able to compensate the loss of function. The major limitation is the prior knowledge of the critical domain of the protein. The strict requirement for the culture medium to avoid leaking expression of Cas9 and the potential dominant negative effect from the inactive protein may also hinder a general application.

Other CRISPR-related gene silencing methods such as CRISPRi, CRISPR-STOP or knockin of an inducible degron tag all face the challenges of the presence of residual protein, which are not discussed here [86–89].

Several algorithms designed to identify splicing motifs could be utilized to avoid unexpected splicing when choosing guide RNAs [23, 90, 91]. Mini-gene assay is also a useful tool to uncover potential splicing regulatory elements [92].

Our favored strategy for dealing with residual protein is RNA interference (RNAi). The inframe transcripts from the disrupted genes are often at low abundance. Therefore, RNAi could efficiently deplete the residual protein to nearly null in knockout cells. The most appreciated advantage of this method is its high convenience, since RNAi is commonly used in research laboratories. Another advantage is the ease of studying protein mutations in such a clean background by simply introducing an RNAi-resistant expression construct together with siRNA oligos in the knockout cells. In case of a lack of the sequence information of the transcript for the residual protein, multiple siRNA oligos targeting distinct mRNA positions need to be tested first. Since most knockout is designed by disrupting the first few exons, siRNA oligo against the downstream sequence could be a good start. However, for abundant transcripts, RNAi may not be able to efficiently deplete the truncated proteins.

## Combining knockout escaping and RNAi for gene function study

**Fig. 3:**
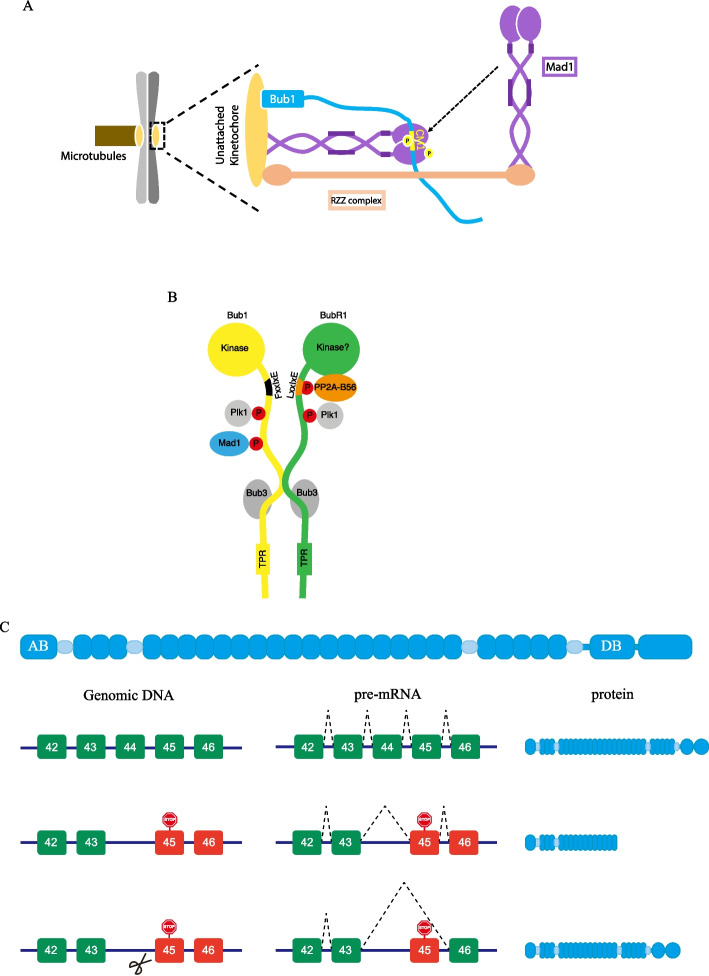
Opportunities resulting from knockout escaping. **A** Combining knockout escaping and RNAi, distinct roles of Bub1 and RZZ on SAC can be dissected. RZZ concentrates Mad1 around Bub1 to enhance Bub1–Mad1 interaction, which is critical for SAC activation [21]. **B** Combining knockout escaping and RNAi reveals the biological significance of the separation of kinase activity and phosphatase activity within the Bub complex. Adapted from Ref. [94]. **C** Knockout escaping provides an opportunity to treat monogenic disease such as DMD. AB (actin binding domain) and DB (b-dystroglycan binding domain) are two essential domains on N-terminal and C-terminal ends. Rounded rectangles with numbers represent exons. Green ones indicate the exons that could be transcribed, and red ones exons that could not be transcribed. Purple lines represent introns. Broken lines indicate the joining sites during the alternative splicing. Scissor means genome editing by CRSPR–Cas. Deletion of exon 44 causes reading frame shift and PTC, resulting in dysfunctional truncated dystrophin. Targeting the splicing acceptor of exon 45 by CRISPR–Cas9 induces exon 45 skipping and restores the reading frame. An internal truncated functional dystrophin is produced and rescues severe DMD [97]

As mentioned above, co-introducing RNAi-resistant constructs and siRNA oligos into knockout cells could enable precise examination of the genuine function of the protein of interest without interference from residual protein. Good examples are from our studies on the mitotic checkpoint proteins Bub1 and Cdc20. Penetrant SAC defect was recorded after we treated *Bub1* knockout cells with siRNA against Bub1. Such a strong phenotype had never been achieved by RNAi treatment in wild-type cells. Introducing exogenous wild-type Bub1 protein fully rescued the SAC defect, confirming the importance of Bub1 in SAC signaling. It was known that both Bub1 and RZZ complex are required for Mad1 kinetochore localization, and the exact role of each was not clear. Similar to Bub1, knocking down any component of RZZ complex by RNAi did not show significant SAC defects, which makes the dissection of Bub1 and RZZ on SAC signaling highly challenging. To solve this problem, we also generated knockout cell lines for Rod, the key component of RZZ complex, with the expression level largely reduced. Complete depletion of either Bub1 or Rod by applying RNAi to the knockout cells reduced the SAC strength to 100 min compared with 600 min in parental HeLa cells after being treated by nocodazole, a tubulin depolymerizing agent. What is more interesting is that artificially tethering Mad1 onto kinetochores bypasses the requirement for Rod, but not Bub1. Therefore, we propose a working model that RZZ concentrates Mad1 onto kinetochores to facilitate the interaction of Bub1 and Mad1, which catalyzes MCC formation [21] (Fig. 3A).

Bub1 is also known to facilitate chromosome alignment during mitosis, with mechanisms that are not fully understood [93]. Using the above system, we precisely measured the contribution of each domain on Bub1 to the chromosome alignment and found that the BubR1 binding domain and the kinase domain were important for the process. Further study revealed that the PP2A/B56 binding motif on Bub1 is degenerated to ensure a robust SAC and that Bub1 facilitates chromosome alignment mainly through the indirectly recruited PP2A/B56 by BubR1. Like the phosphatase activity, spatial separation of the kinase activity in Bub1/BubR1 complex is also required for proper mitosis [94] (Fig. 3B). Adopting the same strategy, our recent study on Cdc20, an essential mitotic regulator, reveals a critical role of the cryptic degron CRY box in checkpoint activation. In the clean background achieved by combining CRISPR–Cas and RNAi, strong SAC defect was recorded in cells complemented with an RNAi-resistant construct expressing Cdc20 without the CRY box. Structural analysis identified several potential interactions among MCC and APC/C components mediated by the CRY box, which were further confirmed by functional and biochemical assays [95].

In general, knockout escaping plus RNAi provides a convenient tool for the study of essential genes where loss of function is difficult to achieve by RNAi alone.

## Knockout escaping for gene therapy

Duchenne muscular dystrophy (DMD) is a fatal muscular degenerative disease with incidence of 1 in 3500–5000 males caused by frameshifting mutations in the gene encoding dystrophin. Dystrophin connects the intracellular cytoskeleton to the extracellular matrix through two essential domains on both termini of the protein (Fig. 3C). The connection protects muscle cell membrane from mechanical damage during muscle contraction. Thus, mutations causing reading frameshift result in dysfunctional protein without the C-terminal domain and cause severe symptoms that mimics the gene knockout phenotype. How could knockout escaping rescue this fatal disease? It is well known that one type of mutation with internal deletions that do not disrupt the reading frame largely maintains the protein functionality and leads to Becker muscular dystrophy (BMD) with mild or even no symptoms. Turning the fatal DMD into mild BMD by skipping exons to restore the reading frame with the help of antisense oligonucleotides has already been approved for clinic treatment. Since the correction occurs at mRNA level, the patients need regular administration during their whole life. To overcome the inconvenience of regular administration, researchers have tried to rescue the fatal mutations with the CRISPR–Cas system [96]. The first attempt was performed by the Akitsu Hotta laboratory. They generated iPSC lines from fibroblasts obtained from a DMD patient with deletion of dystrophin exon 44. Three strategies were tested for correction of the mutated dystrophin in this study. Only the strategy of skipping exon 45 by CRISPR–Cas9 is discussed here. The guide RNA was designed to target the 5′ end of exon 45 with the hope of deleting the splicing acceptor. Indels were successfully introduced into eight clones by CRISPR–Cas9 among 45 clones. Two of the eight clones lost the splicing acceptor site on exon 45. The authors further differentiated the iPSC clones into skeletal muscle cells and examined the mRNA by RT-PCR, which confirmed the skipping of exon 45 and restored the reading frame by the conjugation of exon 43 with exon 46. Immunofluorescence staining of the muscle cells from corrected iPSC clones exhibited strong submembrane dystrophin signals, while no such signals were detected in the muscle cells from DMD iPSC clone. Western blot with an antibody against the C-terminal region also recognized a band of expected size [97]. Thus, the first attempt clearly proved the concept that CRISPR–Cas could be applied for DMD treatment by inducing exon skipping and restoring the reading frame of the remaining exons. Since then, multiple studies have been conducted to correct distinct dystrophin mutations by CRISPR–Cas, achieving promising results in both cells and animals [98–105]. In theory, a similar strategy could be applied to other diseases caused by frameshifting as far as the internal truncated protein could compensate the loss of function to a certain extent.

## Conclusions

Following its first successful application, the CRISPR–Cas system has become the most popular research tool for gene function investigations owing to its high accessibility to general laboratories. Compared with another widely used gene silencing method (RNAi), gene knockout by introducing frameshift via indels is supposed to completely silence the gene. Unfortunately, biological plasticity confers the cells or organisms with certain resistance toward indel-induced gene knockout. Here, we summarize the evidences published in the last few years and show that knockout escaping may be more frequent than realized. The mechanisms leading to knockout escaping are discussed according to several pioneering investigations of knockout and many studies on splicing and translating regulation. We believe that alternative splicing and translation reinitiation but not NAS are the main mechanisms for knockout escaping, which needs to be confirmed in the future by more systematic investigation. Like the warnings already raised in these studies, we strongly recommend a comprehensive characterization of knockout models generated by the CRISPR–Cas system. Even without inframe transcript and residual protein detected, RNAi against the targeted gene could still be applied to knockout models to examine whether a distinct phenotype could be achieved. If that indeed happens, researchers need to consider the possibility of knockout escaping to avoid misinterpreting experimental results. On the other hand, knockout escaping also provides convenient tools for gene function study and monogenic disorder treatment.

## Data Availability

Not applicable.

## Abbreviations

CRISPR: Clustered regularly interspaced short palindromic repeats
Cas: CRISPR-associated protein
DNA: Deoxyribonucleic acid
RNA: Ribonucleic acid
NHEJ: Nonhomologous end-joining
Indels: Insertions and deletions
PTC: Premature termination codon
NMD: Nonsense-mediated decay
NAS: Alternative splicing
ZFN: Zinc-finger nuclease
CDC14: Cell division cycle 14
CK2: Casein kinase 2
CDC37: Cell division cycle 37
Bub1: Budding uninhibited by benzimidazoles 1
BubR1: Bub1 related
Mad1: Mitotic arrest-deficient 1
Cdc20: Cell division cycle 20
RZZ: ROD-Zwilch-ZW10
MCC: Mitotic checkpoint complex
APC/C: Anaphase promoting complex/cyclosome
Bub3: Budding uninhibited by benzimidazoles 3
EpCAM: Epithelial cell adhesion molecule
RT-PCR: Reverse transcription polymerase chain reaction
NGLY1: *N*-Glycanase 1
BRD4: Bromodomain containing 4
DNMT1: DNA methyltransferase 1
TOP1: DNA topoisomerase I
BAC: Bacterial artificial chromosome
KO: Knockout
RHBDF1: Rhomboid 5 homolog 1
Ets2: E26 avian leukemia oncogene 2, 3′ domain
Ctnnb1: Catenin beta 1
WDA1: Wax deficient anther 1
BC10: Brittle culm 10
ATRX: Alpha-thalassemia mental retardation X-linked
BPS: Branch point sequence
PPT: Polypyrimidine tract
ISEs: Intronic splicing enhancers
ISSs: Intronic splicing silencers
ESEs: Exonic splicing enhancers
ESSs: Exonic splicing silencers
RNPs: Ribonucleoproteins
RBPs: RNA binding proteins
ORF: Open reading frame
TCR: T cell receptor
Ig: Immunoglobulin
loxP: Locus of x-over in P1
CRISPRi: CRISPR interference
SAC: Spindle assembly checkpoint
Rod: Rough deal
PP2A/B56: Protein phosphatase 2A/regulatory subunit B56
CRY: Cysteine (C)–arginine (R)–tyrosine (Y)
DMD: Duchenne muscular dystrophy
BMD: Becker muscular dystrophy
iPSC: Induced pluripotent stem cell
